# Plasma cholesterol level determines in vivo prion propagation[S]

**DOI:** 10.1194/jlr.M073718

**Published:** 2017-08-01

**Authors:** Véronique Perrier, Thibaud Imberdis, Pierre-André Lafon, Marina Cefis, Yunyun Wang, Elisabeth Huetter, Jacques-Damien Arnaud, Teresa Alvarez-Martinez, Naig Le Guern, Guillaume Maquart, Laurent Lagrost, Catherine Desrumaux

**Affiliations:** Université Montpellier* and Inserm U1198, Montpellier, F-34095 France and EPHE, Paris, F-75007 France; Cellular Signaling Laboratory,† College of Life Science and Technology, Huazhong University of Science and Technology, Wuhan, Hubei, China; Etablissement Confiné d’Expérimentation A3/L3,§ CECEMA, US009 Biocampus, UMS 3426, Université Montpellier, Montpellier, F-34095 France; INSERM, LNC UMR866,** F-21000 Dijon, France and LNC UMR866, Université Bourgogne Franche-Comté, F-21000 Dijon, France; LipSTIC LabEx,†† Fondation de Coopération Scientifique Bourgogne-Franche Comté, F-21000 Dijon, France; University Hospital of Dijon,§§ F-21000 Dijon, France

**Keywords:** lipid transfer proteins, lipoproteins, neurons, brain, animal models, neurodegenerative diseases, encephalopathy

## Abstract

Transmissible spongiform encephalopathies are fatal neurodegenerative diseases with an urgent need for therapeutic and prophylactic strategies. At the time when the blood-mediated transmission of prions was demonstrated, in vitro studies indicated a high binding affinity of the scrapie prion protein (PrP^Sc^) with apoB-containing lipoproteins, i.e., the main carriers of cholesterol in human blood. The aim of the present study was to explore the relationship between circulating cholesterol-containing lipoproteins and the pathogenicity of prions in vivo. We showed that, in mice with a genetically engineered deficiency for the plasma lipid transporter, phospholipid transfer protein (PLTP), abnormally low circulating cholesterol concentrations were associated with a significant prolongation of survival time after intraperitoneal inoculation of the 22L prion strain. Moreover, when circulating cholesterol levels rose after feeding PLTP-deficient mice a lipid-enriched diet, a significant reduction in survival time of mice together with a marked increase in the accumulation rate of PrP^Sc^ deposits in their brain were observed. Our results suggest that the circulating cholesterol level is a determinant of prion propagation in vivo and that cholesterol-lowering strategies might be a successful therapeutic approach for patients suffering from prion diseases.

Prion diseases are fatal neurodegenerative disorders affecting mammals, and there are neither cures nor palliative therapies to delay their onset or progression (1, 2). They are sporadic, inherited, or acquired disorders. In humans, Creutzfeldt-Jakob disease (CJD) is the most frequent prion disease; while in animals, the most common prion diseases are scrapie, bovine spongiform encephalopathy, and the chronic wasting disease (3, 4). Acquired prion diseases are mostly transmitted through the oral route via the ingestion of contaminated food or via contact with body fluids or feces (5). The hallmark of prion diseases is the deposition of scrapie prion protein (PrP^Sc^), an abnormal β-sheet-rich form of the cellular prion protein (PrP^C^), in the brain (6, 7). Two key events can be targeted to develop therapeutic strategies against transmissible spongiform encephalopathies: first, the conversion of PrP^C^ into the pathological scrapie isoform, PrP^Sc^, which occurs through an autocatalytic process, also called prion replication (8); second, the propagation of PrP^Sc^ from the peripheral circulation to the brain, where it accumulates and causes spongiosis and neurodegeneration (9).

The mechanisms governing prion replication and propagation are not fully elucidated, but evidence suggested that lipids play a role in prion pathogenesis. First, the autocatalytic conversion of the transmembrane protein, PrP^C^, into PrP^Sc^ seems to occur in cholesterol-enriched membrane domains called lipid rafts (10, 11). Phospholipids also act as cofactors of the prion conversion process (12, 13). Regarding prion transport, Safar et al. (14) used a capture-affinity assay to demonstrate that PrP^Sc^ in brain homogenates from CJD patients associates with VLDLs and LDLs, but not with HDLs. This association appears to be mediated by a specific interaction between PrP and apoB, the main protein component of VLDL and LDL particles. These in vitro findings suggest that lipoproteins act as transporters of the prion protein in the peripheral circulation (14).

To establish the in vivo relevance of these observations, we used an established mouse model of dyslipidemia, i.e., phospholipid transfer protein (PLTP)-deficient mice. PLTP is a multifunctional extracellular lipid transport protein involved in lipoprotein metabolism (15–17). PLTP plays a major role in the metabolism of HDLs (18, 19), as well as in the assembly and secretion of apoB-containing lipoproteins in the liver (20) and gut (21). Genetically engineered mice with PLTP deficiency (PLTP^−/−^) constitute a highly relevant model to study the implication of circulating lipids in prion diseases because they display a marked decrease in plasma cholesterol levels and are highly responsive to hypercholesterolizing diets (19, 22). In addition, by contrast with the LDL receptor-deficient or the apoE-deficient mouse models, PLTP deficiency does not modify the uptake of lipids in peripheral cells, which is a prerequisite to address the impact of lipoproteins on prion transport.

We aimed to determine whether manipulation of plasma cholesterol levels in PLTP^−/−^ mice could modulate prion infectivity. Our results showed that PLTP^−/−^ mice fed a standard chow diet exhibited prolonged survival after peripheral prion inoculation due to the slowing down of PrP^Sc^ amyloid deposition and decreased spongiosis in the brain. Remarkably, feeding PLTP^−/−^ mice with a lipid-enriched diet led to a substantial increase in plasma cholesterol and a concomitant reduction in the incubation time after prion inoculation, with more PrP^Sc^ amyloid deposits in animals’ brains. Thus, our data provide the first in vivo evidence of the role of circulating cholesterol levels in prion propagation and suggest that therapeutic strategies aiming at lowering cholesterol levels in plasma might delay disease progression in affected patients.

## MATERIALS AND METHODS

### Animals and ethics statement

PLTP knockout (PLTP^−/−^) and age-matched WT C57BL/6 female mice were used in this study. PLTP^−/−^ mice were provided by Dr. X. C. Jiang (Columbia University, New York) (19). The mice (4–6 weeks old) were housed in an A3/L3 biosafety facility, had free access to water and food, and were fed either a standard chow diet (A03) or a Western-type diet (U8958 version 52) (SAFE Diets).

This project follows the specific French guidelines on animal experimentation and well-being and was approved by the Animal Care and Use Committee Languedoc-Roussillon (Nr. CEEA-LR-12097).

### Biological reagents and antibodies

Pefabloc and proteinase K (PK) were from Roche Diagnostics (Mannheim, Germany). The protein assay kit (BCA) was from Pierce (Thermo Fisher Scientific, Saint Herblain, France). For immunoblotting analyses, we used an anti-prion antibody called SAF84, from SpiBio (Bertin Pharma, Montigny le Bretonneux, France). This antibody recognizes the epitope 161-170 of the PrP proteins. Secondary antibodies were from Jackson ImmunoResearch (West Grove, PA). All other chemicals were from Sigma (Paris, France).

### Prion inoculations

In the first set of experiments, 5 μl of 1% brain homogenate infected with 22L prion strain was inoculated by intracerebral (ic) route into the striatum of PLTP^−/−^ (n = 9) and WT (n = 10) female mice using a stereotaxic frame (Kopf Instruments, Tujunga, CA) and the following coordinates: L, 2.0 mm; R, 0 mm; and P, −3.0 mm (23, 24). For control female mice (n = 10), either PLTP^−/−^ or WT, 5 μl of PBS was inoculated in the striatum using the same method and coordinates. In parallel, transgenic PLTP^−/−^ (n = 5) and WT (n = 5) female mice were intraperitoneally inoculated with 150 μl of 1% brain homogenate infected with 22L prion strain. Control female mice (n = 10), either PLTP^−/−^ or WT, were intraperitoneally inoculated with 150 μl of PBS. Groups of five mice were housed in cages placed in a ventilated protective room. Mice were scored positive for prion disease when three signs of neurologic dysfunction were observed and when progressive deterioration (according to 16 diagnostic criteria) was apparent, as described previously (25, 26). Once clinical signs were detected, the animals were observed daily and euthanized in extremis. Their brains were removed and immediately frozen at −80°C for homogeneization or fixed in AntigenFix (Diapath, France) for immunohistochemical analysis.

In the second set of experiments, groups of PLTP^−/−^ and WT female mice (n = 13 PLTP^−/−^, n = 13 WT) were fed with either a standard chow diet or a Western-type cholesterol-rich diet (Safe diet U8958 version 52) for 4 weeks before being inoculated with 150 μl of 1% brain homogenate infected with the 22L prion strain by the intraperitoneal (ip) route. Then, mice were fed with their respective diets (standard chow or Western-type cholesterol-rich diet). Blood samples for lipid assays were collected by retro-orbital puncture, before (T0) and after the first 4 weeks of the specific diet (standard chow or Western-type cholesterol-rich diet). The mice were observed daily and euthanized in extremis, as described above (25, 26).

### Lipid assays

Freshly drawn blood samples from nonfasted animals were centrifuged at 6,000 *g* for 15 min (4°C). Plasma was collected and kept at −80°C. Total cholesterol and triglycerides were assayed in plasma samples using commercial kits from Diasys (Condom, France); HDL cholesterol was measured using a commercial kit and an Indiko analyzer, both from Thermo Fisher Scientific (Waltham, MA).

### Immunohistochemistry

Brain tissues were fixed in AntigenFix solution (Diapath, France) for 24 h. Then, they were decontaminated for 30 min in formic acid solution according to the protocol described by Andréoletti et al. (27) and stored in 100 mM phosphate buffer at pH 7.4 with 0.02% sodium azide. Samples were dehydrated in graded ethanol, cleared in cedar oil, and embedded in paraffin. Frontal 6 μm sections were cut using a microtome and mounted on Superfrost Plus slides (Microm France, Francheville). Sections were dewaxed and stained with hematoxylin and eosin, as described previously (24). Immunolabeling with anti-GFAP antibodies (1:500; Dako, Les Ulis, France) was performed according to the instructions provided with the Strept ABC Complex kit (Vector Laboratories). Labeling was visualized using 3-3′-diaminobenzidine chromogen solution (Sigma, France). Immunolabeling with anti-Iba-1 antibodies (1:500; Wako Chemicals GmbH, Neuss, Germany) was done after an antigen retrieval step processed by heating glass slides to 100°C in a decloaking chamber (Biocare Medical, Pacheco) for 30 min in citrate buffer (pH 6). The labeling with Iba-1 was then performed as described above using the Strept ABC Complex kit. For paraffin-embedded tissue (PET) blots, 6 μm frontal sections were cut using a microtome and placed onto nitrocellulose membrane. After drying at 50°C for 48 h, sections were dewaxed, digested with 25 μg/ml PK at 56°C overnight and then denatured with 3 M guanidine thiocyanate for 10 min. Membranes were blocked with casein for 30 min. The SAF84 antibody was used to label PrP^Sc^ and the Vectastain ABC-AmP kit (Vector Laboratories) was used to reveal antibody binding. Tissue analysis was performed on several animals (two to three per group, except for asymptomatic animals). For GFAP and Iba-1 immunolabelings, several tissues sections (n = 3–9) were analyzed per group of animals. GFAP-positive cells were quantified by measuring the intensity of the labeling once the background was subtracted, using the Fiji software (2.0 version 2.0; National Institutes of Health, Bethesda, MD) (28). A global threshold was applied to the image for intensity measurement. The values are expressed as mean ± SEM and normalized by surface unit in square millimeters. Iba-1-positive cells were counted using the Fiji software and expressed as mean cell number ± SEM and normalized by surface unit in square millimeters.

### Immunoblotting

Western blot analyses were performed as described previously (29). Briefly, brain tissues were homogenized in 10% (w/v) PBS using microbead-containing tubes and a Ribolyser apparatus (Bio-Rad). Samples were shaken for 45 s and the supernatant was collected through an insulin syringe to obtain a homogeneous suspension. Protein concentrations were measured in each sample using a BCA test (Thermo Fisher Scientific, Illkirch, France) and normalized to have an equivalent level of proteins in each sample before PK digestion test. Fifty microliters of brain homogenates were diluted in 450 μl of PBS with 2% sarcosyl and digested with 20 μg/ml of PK for 1 h at 37°C. The reaction was stopped with 50 μl of Complete Mini (Roche, Switzerland) and 50 μl of each sample were mixed with an equal volume of 2× loading buffer and boiled for 5 min. Thirty microliters were then loaded onto 12% SDS-PAGE precast Criterion gels (Bio-Rad, Marnes-la-Coquette) and analyzed by Western blotting, as described previously (29). PrP^Sc^ was detected with the SAF84 mouse monoclonal antibody, as described previously (24).

### Cytokine assays

Interleukin (IL)-6, IL-1β, and TNF-α were assayed in brain homogenates using ELISA Ready-SET-Go kits from eBiosciences (San Diego, CA) and data were normalized to total protein concentrations.

### Software and statistical analyses

Kaplan-Meier survival curves were done using the GraphPad Prism software (La Jolla, CA). Animals inoculated by ip route and fed the chow diet (first and second set of experiments) were analyzed as a single group. The difference between curves was tested using the nonparametric Mantel-Cox test, with a probability of 0.05 defined as a significant difference. Survival times are expressed as median values and lipid levels are expressed as mean ± SEM. The statistical significance of differences between data means was determined with a Student’s *t*-test or a two-way ANOVA analysis, as appropriate.

## RESULTS

### Plasma lipid profiles in PLTP^−/−^ and WT mice fed a standard diet

**Fig. 1.**
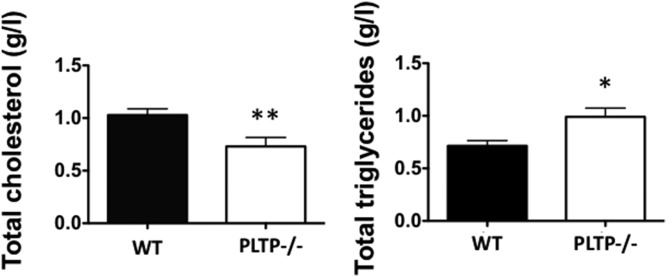
PLTP^−/−^ mice present a 30% decrease in plasma cholesterol compared with WT animals. Total cholesterol and triglycerides were assayed in plasma samples from WT (n = 8) and PLTP^−/−^ mice (n = 8) before their inoculation with prions, using commercial kits from Diasys. Results are expressed in grams per liter and correspond to mean ± SEM. *P < 0.05, **P < 0.01, Student’s *t*-test.

Prior to prion inoculation, cholesterol and triglyceride levels were measured in the plasma of PLTP^−/−^ and WT mice fed a standard diet. As previously reported (18, 19, 30), PLTP^−/−^ mice showed abnormal lipid levels in plasma, with a significantly lower total cholesterol concentration (0.72 ± 0.09 g/l vs. 1.01 ± 0.06 g/l in WT mice, *P* < 0.01) (**Fig. 1**, left panel) and a significantly higher triglyceride concentration (1.00 ± 0.09 g/l vs. 0.72 ± 0.05 g/l in WT mice, *P* < 0.05, Student’s *t*-test) (Fig. 1, right panel) compared with WT animals.

### Survival of chow diet-fed PLTP^−/−^ and WT mice after ic or ip inoculation with prions

**Fig. 2.**
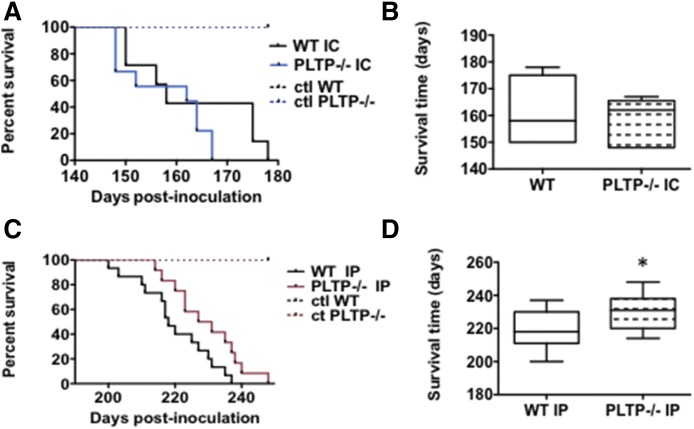
PLTP^−/−^ mice challenged intraperitoneally with prions exhibited prolonged survival compared with WT counterparts. A: Kaplan-Meier survival plots of WT (in black, n = 10) and PLTP^−/−^ (in blue, n = 9) mice inoculated intracerebrally with 5 μl of brain homogenate infected with 22L prion strain. In each group (WT and PLTP^−/−^), five mice were inoculated with 5 μl of PBS by ic (IC) route to serve as controls (ctl). B: Box-and-whiskers representation of median survival time of plots presented in (A). The median survival time, 158 dpi for WT mice versus 162 dpi for PLTP^−/−^ mice, is not statistically different [Log-rank (Mantel-Cox) test]. C: Kaplan-Meier survival plots of WT (in black, n = 15) and PLTP^−/−^(in red, n = 13) mice inoculated intraperitoneally with 150 μl of brain homogenate infected with 22L prion strain. In each group, five mice were inoculated with 150 μl of PBS by the ip route to serve as controls. D: Box-and-whiskers representation of median survival time of plots presented in (C). The median survival time, 218 dpi for WT mice versus 231 dpi for PLTP^−/−^ mice, is statistically different [*P < 0.05, Log-rank (Mantel-Cox) test].

Because PLTP^−/−^ mice exhibit reduced cholesterol levels in plasma, but no difference in cholesterol levels in the brain (19, 31, 32), we chose to evaluate the impact of PLTP deficiency on prion propagation using two different routes of administration, i.e., ic and ip. Control animals, which had been inoculated with PBS, remained healthy during the whole experiment. As shown in **Fig. 2**, WT and PLTP^−/−^ mice inoculated with the 22L prion strain through the ic route had comparable survival curves [median survival: 158 days postinjection (dpi) for WT mice vs. 162 dpi for PLTP^−/−^ mice, NS, Log-rank (Mantel-Cox) test] (Fig. 2A, B). In contrast, survival in the PLTP^−/−^ group challenged with prion by the ip route was significantly longer than that in their WT counterparts [median survival time, 218 dpi for WT mice vs. 231 dpi for PLTP^−/−^ mice, *P* < 0.05, Log-rank (Mantel-Cox) test] (Fig. 2C, D).

### Impact of a Western diet on plasma lipid profiles in WT and PLTP^−/−^ mice

**Fig. 3.**
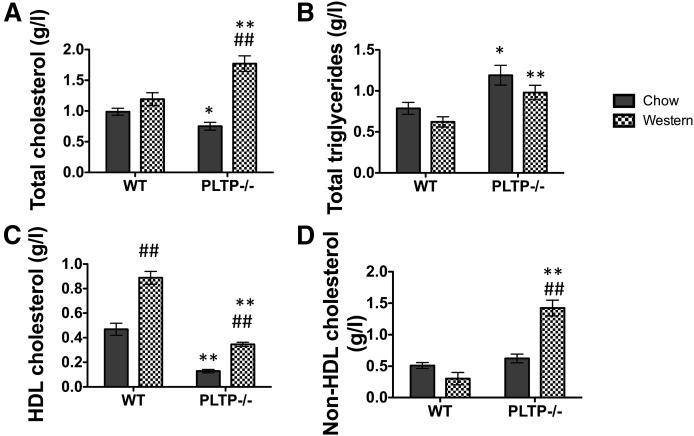
Western-type cholesterol-rich diet substantially increases plasma levels of cholesterol in PLTP^−/−^ mice. Total cholesterol (A), triglycerides (B), and HDL-cholesterol (C)were assayed in plasma samples from WT (n = 8) and PLTP^−/−^ (n = 8) mice after they had been fed for 4 weeks either with a standard chow diet or a Western-type diet. Non-HDL cholesterol was calculated as the difference between total and HDL-cholesterol (D). Results are expressed in grams per liter and correspond to mean ± SEM (*P < 0.05,**P < 0.001 vs. WT, Student’s *t*-test; ^#^*P* < 0.05, ^##^ *P* < 0.001 vs. chow, two-way ANOVA analysis with Bonferroni’s post hoc analysis).

Because the reduced levels of cholesterol in PLTP^−/−^ mice were associated with prolonged survival after ip prion inoculation, we hypothesized that elevating plasma cholesterol levels through a specific lipid-enriched diet might facilitate prion propagation. Mice were fed either a standard chow diet or a Western-type lipid-enriched diet for 4 weeks before prion ip inoculation (**Fig. 3A**). Lipid assays were performed on plasma samples from nonfasted animals, collected before and 4 weeks after they were put on their specific diet. A nonsignificant increase in plasma total cholesterol was observed in WT animals fed the Western-type diet compared with the chow diet (1.19 ± 0.11 g/l vs. 0.98 ± 0.06 g/l, respectively); while in PLTP^−/−^ mice, plasma total cholesterol raised above the normal level with a strong 135% increase in the Western diet-fed group (1.77 ± 0.13 g/l vs. 0.75 ± 0.06 g/l in the chow diet-fed group, *P* < 0.001, Student’s *t*-test) (Fig. 3B). Two-way ANOVA analysis of plasma total cholesterol levels showed a statistical effect for the diet [F(1,25) = 40.34, *P* < 0.001] and the interaction diet × genotype [F(1,25) = 17.85, *P* < 0.001]. We further determined cholesterol distribution between HDL and non-HDL particles. In mice fed the standard chow diet, a dramatic 79% decrease in HDL-cholesterol, but not in non-HDL-cholesterol, was measured in PLTP^−/−^ mice compared with WT mice (*P* < 0.001). In Western diet-fed animals, HDL-cholesterol levels increased in similar proportions in both WT and PLTP^−/−^ mice, while non-HDL-cholesterol was markedly increased only in PLTP^−/−^ mice (Fig. 3C, D).

The Western diet did not significantly modify the levels of triglycerides in WT or PLTP^−/−^ mice compared with animals fed the chow diet. Two-way ANOVA analysis of plasma total triglyceride levels showed a statistical effect for the genotype only [F(1,26) = 18.75, *P* < 0.001], because the PLTP^−/−^ genotype significantly increased the levels of triglycerides compared with WT animals, either on the chow or the Western-type diet.

### Impact of Western-type diet on survival time in WT and PLTP^−/−^ mice after ip prion inoculation

**Fig. 4.**
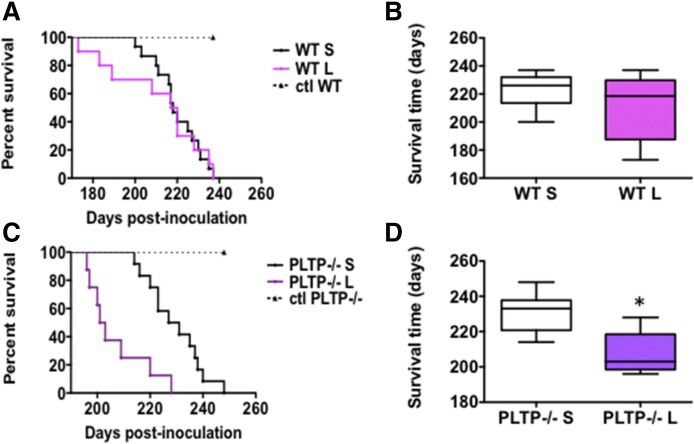
Western-type cholesterol-rich diet markedly accelerates prion disease in PLTP^−/−^ mice. A: Kaplan-Meier survival plots of WT mice inoculated with 150 μl of 22L prion strain by the ip route and fed with either a standard chow diet (WT S; in black, n = 15) or a Western-type cholesterol-rich diet (WT L; in pink, n = 10). In each group (WT S and WT L), five mice were inoculated with 150 μl of PBS by the ip route to serve as controls. B: Box-and-whiskers representation of median survival time of Kaplan-Meier plots presented in (A). The median survival time, 226 dpi for WT S mice versus 218 dpi for WT L mice, is not statistically different [Log-rank (Mantel-Cox) test]. C: Kaplan-Meier survival plots of PLTP^−/−^ mice inoculated with 150 μl of 22L prion strain by the ip route and fed with either a standard chow diet (PLTP^−/−^ S; in black, n = 13) or a Western-type cholesterol-rich diet (PLTP^−/−^ L; in purple, n = 10). In each group (PLTP^−/−^ S and PLTP^−/−^ L), five mice were inoculated with 150 μl of PBS by the ip route to serve as controls. D: Box-and-whiskers representation of median survival time of Kaplan-Meier plots presented in (C). The median survival time, 233 dpi for PLTP^−/−^ S mice versus 203 dpi for PLTP^−/−^ L mice, is statistically significant (*P < 0.05, Mantel-Cox test).

Both WT and PLTP^−/−^ control animals that had been inoculated with PBS remained healthy during the whole experiment. The survival curves of WT mice were not significantly different whether the animals were fed the chow or the Western-type lipid-enriched diet (median survival, 226 dpi on the chow diet vs. 218 dpi on Western diet) (**Fig. 4A, B**). In contrast, a substantial decrease of the survival time was observed in PLTP^−/−^ mice fed the Western-type diet compared with the group fed the chow diet, and the profile of the curves was well-separated [median survival time, 233 dpi with the chow diet vs. 203 dpi with the Western diet; *P* < 0.002, Log-rank (Mantel-Cox) test] (Fig. 4C, D).

### Detection of the PrP^Sc^ marker by Western blot in brains of WT and PLTP^−/−^ mice

**Fig. 5.**
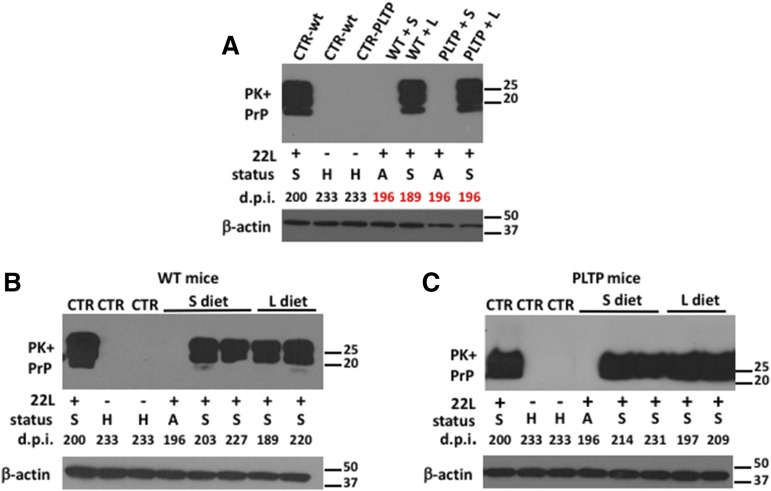
Detection of the pathologic PrP^SC^ marker in brain homogenates of asymptomatic and sick mice. A–C: Brain homogenates of WT or PLTP^−/−^ mice, inoculated (+) or not (−) with 22L prion strain and fed a standard chow diet (+S) or Western-type cholesterol-rich diet (+L) were analyzed by immunoblotting after PK digestion (PK+). The numbers below each panel indicate the day when the mice were euthanized (dpi). The letters below each panel (S, sick; H, healthy; A, asymptomatic) indicate the status of the mice when they were euthanized. PrP means that the immunoblots were probed with SAF84 anti-PrP antibodies. Loading control blots, performed on the identical samples before PK digestion were probed with anti-β-actin antibodies. The molecular weight markers are indicated on the right side of each immunoblot.

After partial PK digestion to eliminate PrP^C^, brain homogenates were subjected to Western blot analysis using the SAF84 anti-PrP antibody to detect the presence of PrP^Sc^ (29). As shown in **Fig. 5A**, no signal corresponding to PrP^Sc^ mon­omers was observed in homogenates from noninoculated healthy mice. The first mice that presented the typical symptoms of the disease were those fed the Western-type lipid-enriched diet. When the first PLTP^−/−^ mice in this group needed to be euthanized (196 dpi), WT and PLTP^−/−^ mice in the groups fed the chow diet were also euthanized on the same day (196 dpi), even though they were still asymptomatic (for Western blot and histological analysis), in order to assess prion levels in their brains. Western blot analysis revealed no detectable PrP^Sc^ in the brains of asymptomatic WT and PLTP^−/−^ mice at 196 dpi (Fig. 5A). In contrast, a substantial amount of PrP^Sc^ was detected in PLTP^−/−^ and WT mice fed the Western-type lipid-enriched diet and euthanized at 196 dpi and 189 dpi, respectively (Fig. 5A). Brain homogenates from mice fed either the chow or the Western-type diet and euthanized at the end-stage of the disease contained high and equivalent amounts of PrP^Sc^, whatever their genotype (Fig. 5B for WT mice and Fig. 5C for PLTP^−/−^ mice).

### Histological assessment of prion propagation in brain tissue of WT and PLTP^−/−^ mice

**Fig. 6.**
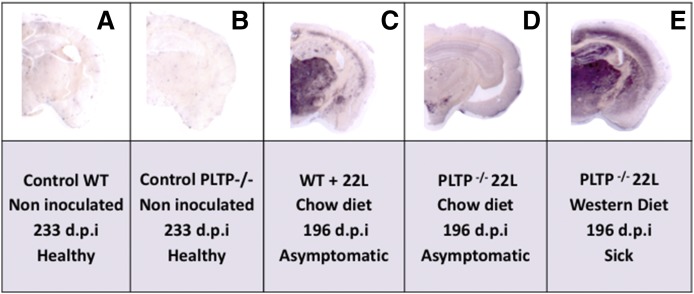
The PrP^Sc^ amyloid load is substantially decreased in PLTP^−/−^ mice fed a standard chow diet. A, B: PET blot analyses of frontal tissue sections of WT or PLTP^−/−^ mice not inoculated with prions and euthanized at 233 days (dpi) while they were healthy. C–E: PET blot analyses of frontal tissue sections of WT or PLTP^−/−^ mice inoculated with 22L prion strain, fed a standard chow diet or Western-type cholesterol-rich diet, and all euthanized on the same day (196 dpi), when they were in an asymptomatic stage (C, D) or while they were at the terminal stage of the disease (E). The SAF84 antibody was used to detect PrP^SC^ proteins and the Vectastain ABC-AmP kit (Vector Laboratories) was used to reveal antibody binding.

Because we did not find detectable levels of PrP^Sc^ in brain homogenates from asymptomatic WT and PLTP^−/−^ mice, we further searched for the presence of the PrP^Sc^ marker and assessed its regionalization by histological analysis using the PET blot technique. A substantial difference in prion accumulation was revealed between asymptomatic PLTP^−/−^ and WT mice on PET blots (**Fig. 6**). At 196 dpi, PrP^Sc^ was barely detectable in the brain of asymptomatic PLTP^−/−^ mice (Fig. 6D); while in asymptomatic WT mice, PrP^Sc^ deposits were observed in the thalamus and started to appear in the cortex (Fig. 6C), and in Western diet-fed sick PLTP^−/−^ mice, abundant PrP^Sc^ deposits were also present in the cortex, the hippocampus, and striatal regions of the brain (Fig. 6E). Noninoculated WT and PLTP^−/−^ animals did not present any PrP^Sc^ deposits in the brain (Fig. 6A, B). No difference was observed at the terminal stage of the disease (supplemental Fig. S1).

### Assessment of brain inflammation in WT and PLTP^−/−^ mice

**Fig. 7.**
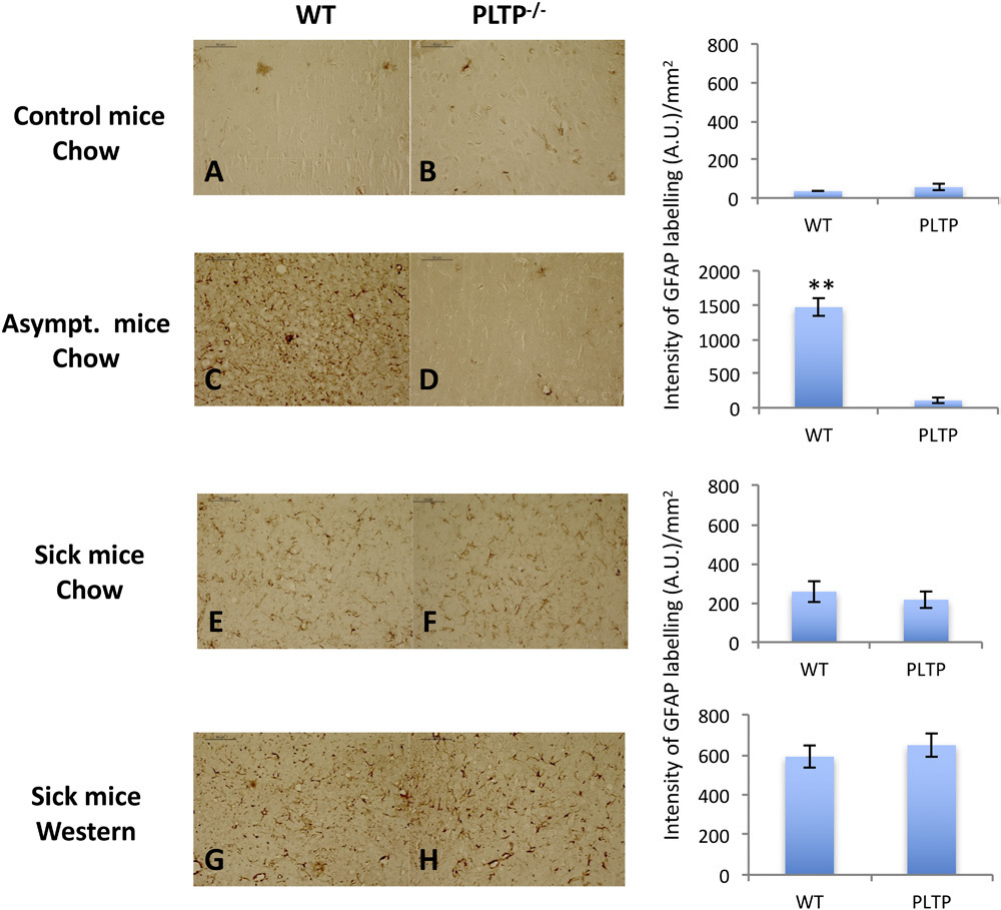
Analysis of astrogliosis in brain tissue sections. A, B: Histological analyses of thalamus sections from healthy control WT and PLTP^−/−^ mice not inoculated with prions. C–H: Thalamus tissue sections from WT and PLTP^−/−^ mice inoculated with 22L prion strain and either euthanized while they were asymptomatic (196 dpi) (C, D) or at the terminal stage of the disease (E–H), and fed either a standard chow diet (E, F) or a Western-type cholesterol-rich diet (G, H). Tissue sections were stained using an anti-GFAP antibody to detect astrocytic gliosis, a marker of the inflammation process during prion disease. Tissue labeling was performed on several animals (two to three per group, except asymptomatic mice) and the selected images (enlargement ×20) are representative of the staining observed in each group. The intensity of the GFAP labeling was used to quantify the number of GFAP-positive cells and is expressed as mean intensity ± SEM (arbitrary units per square millimeter). Student’s *t*-test was used for statistical data analyses (**P < 0.01).

In prion diseases, astrogliosis is a marker of brain inflammation consecutive to the neuroinvasion process. Differences of astrogliosis in PLTP^−/−^ mice and WT animals could have an impact on animals’ survival time. Thus, we performed histopathological analyses in healthy noninoculated animals as negative controls to evaluate the astrogliosis status of PLTP^−/−^ mice compared with WT animals (**Fig. 7A, B**). We also assessed astrogliosis in WT and PLTP^−/−^ animals after 22L prion inoculation, either at the asymptomatic (Fig. 7C, D) or terminal stage of the disease (Fig. 7E–H). Astrocytic gliosis was analyzed using anti-GFAP antibodies with colorimetric detection. Astroglial activation was barely detected in noninoculated healthy mice and no statistically significant difference was observed between WT and PLTP^−/−^ animals (Fig. 7A, B). In asymptomatic mice, a higher level of reactive astrocytes was observed in WT compared with PLTP^−/−^ mice (Fig. 7C, D) euthanized on the same day. This observation corroborates the PET blot analysis, showing higher PrP^Sc^ levels in WT mice than in PLTP^−/−^ mice (Fig. 6C, D). This illustrates the advanced neuroinvasion process that occurred in the WT animals with a strong astrocytic gliosis in response to the prion invasion, which was not yet the case for the PLTP^−/−^ mice. At the terminal stage of the disease, all groups exhibited increased levels of astrogliosis (Fig. 7E–H) compared with noninoculated animals, which is a typical feature of prion sick animals, as described previously (24). The astrogliosis levels were equivalent between the genotypes (Student’s *t*-test, *P* > 0.05) (Fig. 7E vs. Fig. 7F and Fig. 7G vs. Fig. 7H). Interestingly, one can notice that the intensity of astrogliosis was doubled (Fig. 7G, H) under the Western diet in both genotypes, compared with animals fed the chow diet (Fig. 7E, F). Altogether, these data show that both genotypes display equivalent levels of astrogliosis, thus this criterion is unlikely to be implicated in the survival time differences between WT and PLTP^−/−^ animals.

**Fig. 8.**
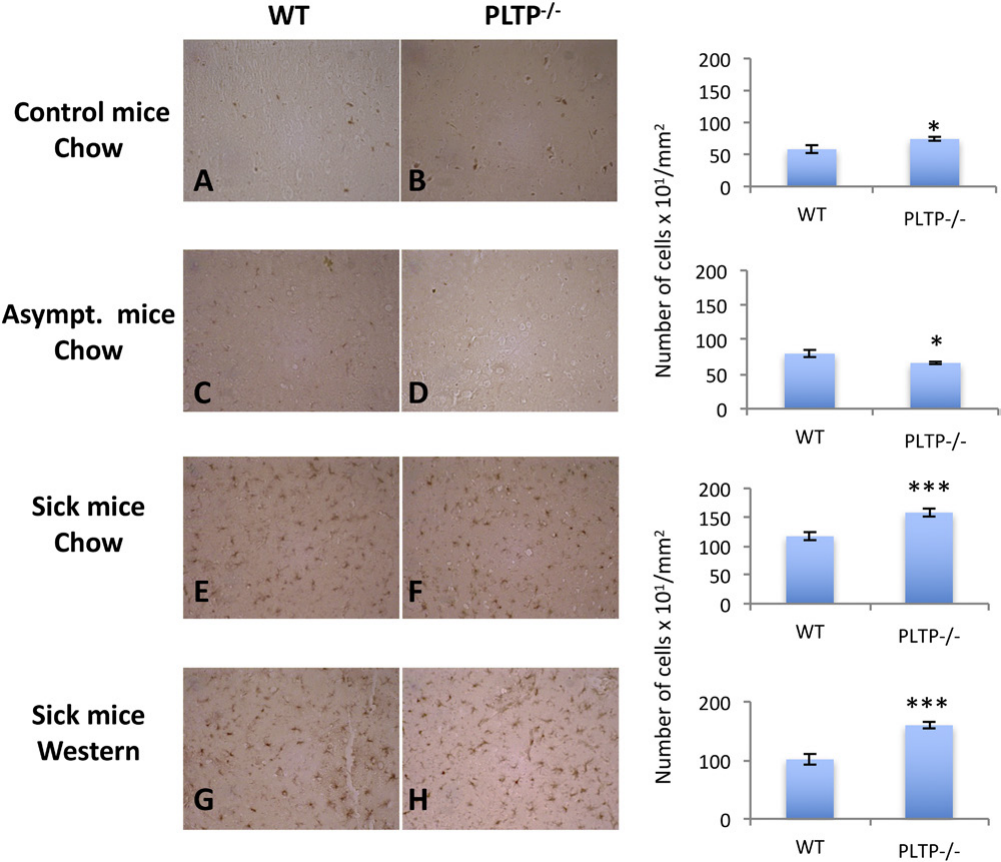
Analysis of microglia in brain tissue sections. A, B: Histological analyses of thalamus sections from control WT and PLTP^−/−^ mice not inoculated with prions. C–H: Thalamus tissue sections from WT and PLTP^−/−^ mice inoculated with 22L prion strain and either euthanized while they were asymptomatic (196 dpi) (C, D) or at the terminal stage of the disease (E–H), and fed either a standard chow diet (E, F) or a Western-type cholesterol-rich diet (G, H). Tissue sections were stained with an anti-Iba1 antibody to detect microglia, a marker of brain inflammation during neurodegenerative disease. Tissue labeling was performed on several animals (two to three per group, except asymptomatic mice) and the selected images (enlargement ×40) are representative of the staining observed in each group. Quantification of Iba-1-positive cells is expressed as the mean ± SEM of the number of cells per square millimeter. Student’s *t*-test was used for statistical data analyses (*P <0.05, ***P < 0.001).

We also checked the microglial response in WT and PLTP^−/−^ mice (**Fig. 8**). The role of microglial cells in prion pathogenesis seems to depend on disease stage. Microglia would be neuroprotective at the early stage of the infection through their ability to clear PrP^Sc^; however, it has been shown that once microglia numbers are upregulated during the course of the disease, they do not exhibit efficient prion clearing capacity and promote neuroinflammation (33). To decipher the role of microglia and their implication in prion survival time associated with PLTP deficiency, tissue sections were labeled with an anti-Iba-1 antibody and positive cells were counted using ImageJ. The brain tissue sections revealed a significant increase of 27% in micro­glial cell numbers in healthy noninoculated PLTP^−/−^ mice compared with WT animals (microglial cells per square millimeter, mean ± SEM: 7,430 ± 300 vs. 5,830 ± 610 in WT, *P* < 0.05, Student’s *t*-test) (Fig. 8A, B). At the terminal stage of the disease, we also observed a 35% increase of microglial cells in PLTP^−/−^ mice in the chow diet-fed group (1,581 ± 68 vs. 1,171 ± 69 in WT, *P* < 0.001, Student’s *t*-test) (Fig. 8E, F). The effect was even more drastic for groups fed the Western-type diet, with an increase of 58% of microglial cells in PLTP^−/−^ versus WT animals (1,601 ± 55 vs. 1,016 ± 90 in WT, *P* < 0.001, Student’s *t*-test) (Fig. 8G, H).

**Fig. 9.**
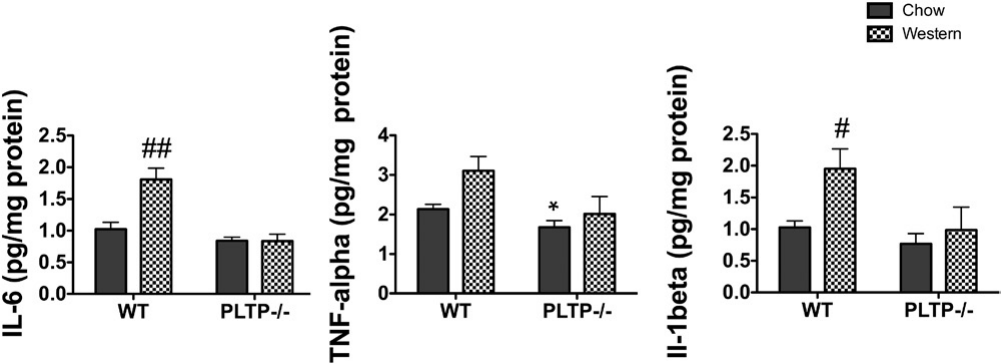
Cytokine levels in WT and PLTP^−/−^ mouse brains fed a standard chow diet or a Western-type diet. IL-6, TNF-α, and IL-1β were assayed in brain homogenates from WT and PLTP^−/−^ mice (n = 7–10) after they had been fed for 4 weeks either with a standard chow diet or a Western-type cholesterol-rich diet. Results correspond to mean ± SEM (*P < 0.05 vs. WT, Student’s *t*-test; ^#^*P* < 0.05, ^##^*P* < 0.001 vs. chow, two-way ANOVA analysis with Bonferroni’s post hoc analysis).

To further address the potential impact of brain inflammatory state on prion disease in WT and PLTP^−/−^ mice, the prototypic pro-inflammatory cytokines, IL-6, IL-1β, and TNF-α, were assayed in brain homogenates from mice fed the standard chow or the Western diet. As shown in **Fig. 9**, in mice fed the standard chow diet, IL-6 and IL-1β levels were mildly, but not significantly, decreased in PLTP^−/−^ mouse brains compared with WT, while a significant 27% decrease was measured for TNF-α (*P* < 0.05). Upon Western diet feeding, IL-6 and IL-1β levels were markedly increased in WT mouse brains (+76% and +89%, respectively, *P* < 0.05), while they remained constant in PLTP-deficient tissue. In a similar way, TNF-α levels increased by 45% in WT mouse brains (NS) and remained constant in PLTP-deficient mouse brains.

### Histological assessment of spongiosis in the brain tissue of WT and PLTP^−/−^ mice

Hematoxylin and eosin staining of paraffin-embedded tissue sections was performed to assess spongiosis in the tissue. Spongiosis is another marker of prion diseases, reflecting the neuronal loss by the presence of numerous white and round vacuoles in the brain of sick animals. As expected, no spongiosis was observed in noninoculated healthy mice (supplemental Fig. S2A, B). In mice euthanized while they were still asymptomatic, very few small vacuoles were observed in tissue sections from both WT and PLTP^−/−^ animals (supplemental Fig. S2C, D). In contrast, extensive tissue spongiosis was present in all groups euthanized at the terminal stage of the disease and presenting classical prion symptoms (supplemental Fig. S2E–H).

## DISCUSSION

Most animal and acquired human prion diseases are mainly due to oral or peripheral contamination (5). Thus, understanding the molecular mechanisms involved in prion propagation could lead to the identification of new markers or key steps in the disease process that might help to prevent prion agents from propagating to and replicating in the brain. Several routes of transmissible spongiform encephalopathy propagation from the peripheral system to the CNS have been identified. One of them is by spreading in a retrograde direction along efferent fibers of both sympathetic and parasympathetic nerves (34, 35). In parallel, the neuroinvasion process can also occur through the bloodstream, independently of the peripheral nervous system (36, 37). Experimental transmission studies conducted in ovines and primates indicated that 30% of prion infectivity is associated with white blood cells and 50% with the plasma fraction, while total blood remains the most infectious (38, 39). In 2004, with the appearance of variant CJD cases through contaminated blood transfusion, this mode of transmission was demonstrated in humans (40, 41). The identification of the macromolecules implicated in prion transport via the bloodstream is a challenge for the development of therapeutic strategies. Interestingly, at the time when the blood-mediated transmission route was demonstrated, in vitro studies published by Safar et al. (14) indicated a high binding affinity of the PrP^Sc^ with apoB-containing lipoproteins, i.e., the main carriers of cholesterol in human blood. Thus, our aim was to determine the pathophysiological relevance of these findings and to address the role of circulating cholesterol-containing lipoproteins in the pathogenicity of prions in vivo. We took advantage of the PLTP-deficient mouse model. As described earlier, PLTP deficiency in mice is associated with a marked decrease in HDL-cholesterol levels, due to hypercatabolism and defective production of apoB-containing lipoprotein particles by the liver and intestine (18–20), which together contribute to abnormally low circulating cholesterol concentrations (Fig. 1); in contrast, cholesterol contents of peripheral tissues, including the brain, remain unaffected by PLTP deficiency (19, 20, 31, 32).

We showed that the PLTP-deficient trait was associated with significantly prolonged survival after ip inoculation of the 22L prion strain (Fig. 2C). It was accompanied by a reduction in the rate of accumulation of PrP^Sc^ aggregates in brain tissue (Figs. 5, 6). Moreover, and in support of a direct cause-effect relationship between high plasma cholesterol concentrations and accumulation of PrP^Sc^ aggregates, increasing circulating cholesterol levels by the means of dietary manipulation produced a significant reduction in survival times in PLTP^−/−^ mice, as well as a marked increase in the accumulation rate of PrP^Sc^ deposits in their brains (Figs. 4C, 6D, E). The relationship between circulating cholesterol levels and prion propagation is further reinforced by the fact that, in WT mice, the lipid-enriched diet did not lead to a significant rise in circulating cholesterol levels, as these mice have better regulation of cholesterol metabolism, and no statistically significant difference was observed in survival times (Fig. 4A). To address the question of whether all lipoprotein particles or only a specific subpopulation of them contribute to prion transport, we measured plasma HDL-cholesterol and non-HDL-cholesterol levels. In mice fed the standard chow diet, a dramatic 79% decrease in HDL-cholesterol, but not in non-HDL-cholesterol, was measured in PLTP-deficient mice compared with WT mice (Fig. 3C), suggesting that, in the context of standard diet feeding, HDL-containing lipoproteins play a key role in peripheral prion transport and propagation to the brain in our mouse model. In Western diet-fed animals, in agreement with previous reports (42), HDL-cholesterol levels increased in similar proportions in both WT and PLTP^−/−^ mice, while non-HDL-cholesterol was markedly increased only in PLTP^−/−^ mice (Fig. 3D); because PLTP is known to play a major role in lipid transfers from non-HDL to HDL particles during the postprandial state (15), this discrepancy most probably relies on the inefficient clearance of non-HDL particles in PLTP-deficient animals. The marked decrease in PLTP^−/−^ mouse survival in the Western diet-fed group compared with the chow diet-fed group suggests that non-HDL-containing lipoproteins play a critical role in prion transport, which is in good agreement with the data reported by Safar et al. (14) showing that PrP^Sc^ in sporadic CJD patients associates with VLDLs and LDLs.

Taken together, our data show that both HDL and non-HDL particles can act as transporters for PrP^Sc^ molecules, but only total plasma cholesterol can be used to evaluate the extent of prion propagation from the periphery to the brain, and predict survival time. In contrast to cholesterol, triglycerides were unlikely to affect prion propagation because they were not statistically modified by the lipid-enriched diet, whatever the genotype of the mice (Fig. 3B).

It is noteworthy that due to reduced circulating levels of cholesterol-containing lipoproteins in PLTP^−/−^ mice, the prion protein is less efficiently transported to the brain and may be more rapidly cleared from the blood by mononuclear phagocytes (dendritic cells and macrophages) (9).

Membrane cholesterol also plays an important role in prion conversion. The structure and integrity of lipid rafts, i.e., membrane domains enriched in cholesterol and sphingomyelin, are essential for the conversion of PrP^C^ into PrP^Sc^ (10, 11). Moreover, raft disruption through cholesterol depletion decreases PrP^Sc^ formation (43–46). In our PLTP-deficient mouse model, brain cholesterol levels, in contrast to circulating cholesterol levels, were not modified (31, 32) and, accordingly, when WT and PLTP^−/−^ mice were inoculated with prions by the ic route, no difference in their survival time was observed (Fig. 2A).

Besides cholesterol lowering, PLTP deficiency is associated with a low inflammatory state in the periphery (47–50) that may also play a role in the observed effect on prion pathogenesis. Regarding brain inflammation, no data have been reported so far. In the present study, while the survival time was clearly reduced in PLTP^−/−^ mice compared with WT mice under the chow diet, brain astrogliosis (as assessed by GFAP labeling) was similar in both genotypes either in control noninoculated animals or at the terminal stage of the disease, whatever the diet (Fig. 7A, B, E–H). Thus, this criterion is unlikely to be implicated in the survival time differences between WT and PLTP^−/−^ animals. It is worthy of note that at the beginning of the neuroinvasion process, a strong astrogliosis occurs to protect neurons against prions (as observed in WT asymptomatic mice, Fig. 7C). However, at the terminal stage of the disease, when prion propagation is completed, less astrogliosis is observed because astrocytes are dying due to the appearance of numerous PrP^Sc^ deposits and brain spongiosis that are detrimental to them (Fig. 7E).

We also checked the microglia response (Fig. 8) in WT and PLTP^−/−^ mice. The role of microglial cells in prion pathogenesis seems to depend on disease stage. Microglia seem to be neuroprotective at the early stage of the infection through their ability to clear PrP^Sc^; however, it has been shown that once microglia numbers are upregulated during the course of the disease, they do not exhibit efficient prion clearing capacity and promote neuroinflammation (33). The brain tissue sections revealed a significant increase in microglial cell numbers in healthy noninoculated PLTP-deficient mice compared with WT mice. The same observation was made at the terminal stage of the disease, whether the mice were fed the standard chow diet or the Western diet. Because microglial cells are able to clear PrP^Sc^ at the early stage of the disease and can have a neuroprotective role (33), we would have expected that a basal increase of 27% in PLTP^−/−^ mice would lead to higher survival time of animals intracerebrally challenged with prions. However, it was not the case (Fig. 2). By opposition, if microglia had promoted a deleterious neuro-inflammatory response under our experimental conditions, then we should have observed a decrease rather than an increase in the survival time of PLTP^−/−^ mice fed the standard chow diet. For these reasons, it seems very unlikely that microglial cell numbers are related to survival times in our study.

To further address the potential impact of brain inflammatory state on prion disease, the prototypic pro-inflammatory cytokines, IL-6, IL-1β, and TNF-α, were assayed in brain homogenates from WT and PLTP^−/−^ mice fed the standard chow or the Western diet. The levels of all three cytokines remained unchanged in PLTP^−/−^ mice fed either with the standard chow or the Western diet, despite a significant reduction of their survival time; in WT mice, a strong increase in brain cytokines occurred in the Western diet-fed group, although their survival time remained unchanged; taken together, these data argue against a role of brain cytokine levels in prion propagation in our model.

Altogether, the results of the present study strongly support key participation of circulating cholesterol-rich lipoproteins in the propagation of prion agents from the peripheral system to the brain. PLTP^−/−^ mice exhibit prolonged survival compared with WT animals, but this phenotype is reversed when animals are fed a hypercholesterolizing diet. Our study suggests that targeting circulating cholesterol may be a relevant therapeutic strategy. In previous studies, mice treated with the cholesterol-lowering drug, simvastatin, exhibited a substantial increase in their survival time (51, 52). However, the authors suggested that it might be linked to pleiotropic effects of statins, especially their immunomodulatory and anti-inflammatory properties. Although no data on plasma cholesterol are available in the above-mentioned studies, the increased survival time of simvastatin-treated mice might be explained, in part, by a reduction of plasma cholesterol level (53), as observed in our PLTP^−/−^ mice.

## CONCLUSIONS

Our results suggest that the circulating cholesterol level is a determinant of prion propagation in vivo and combining strategies to lower circulating cholesterol and restore cellular cholesterol homeostasis with inhibitors of cholesterol esterification enzymes could be a successful approach to treat prion diseases.

## Supplementary Material

Supplemental Data
