# Supplementary material for: Plasma cholesterol level determines in vivo prion propagation
Source: J Lipid Res. 2017 Aug 1;58(10):1950–61. doi: 10.1194/jlr.M073718 (PMC5625119; doi:10.1194/jlr.M073718)
Supplement: Supplemental Data [file supp_58_10_1950__index.html]

Plasma cholesterol level determines in vivo prion propagation — Plasma cholesterol level determines in vivo prion propagation — Plasma cholesterol level determines in vivo prion propagation — Supplemental Data 

# Plasma cholesterol level determines in vivo prion propagation

## Supplemental Data

- Supplemental Figure S1 (.pdf, 427 KB) - Supplemental Figure S1
- Supplemental Figure S2 (.pdf, 468 KB) - Supplemental Figure S2
