# Supplementary material for: Plasma cholesterol level determines in vivo prion propagation
Source: J Lipid Res. 2017 Aug 1;58(10):1950–61. doi: 10.1194/jlr.M073718 (PMC5625119; doi:10.1194/jlr.M073718)
Supplement: Supplemental Data [file 10.1194_M073718_jlr.M073718-1.pdf]

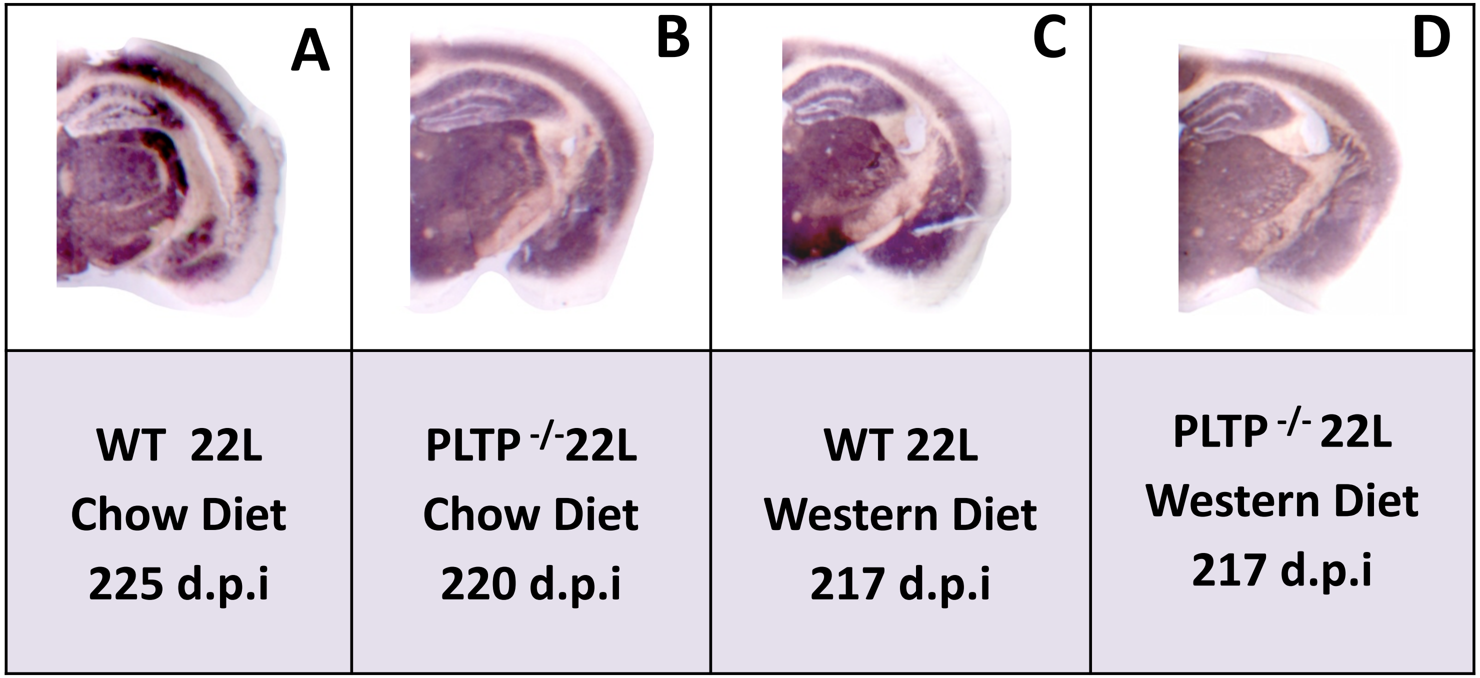

Supplemental Figure S1. Immunoblotting of tissue sections of PLTP$^{-/-}$ and WT mice, killed at the terminal stage of the disease.
PET-blot analysis of frontal tissue sections from WT or PLTP$^{-/-}$ mice inoculated with 22L prions and sacrificed while they were sick, and presenting equivalent incubation times of the disease (217, 220, 225 d.p.i). Mice were fed a standard chow diet (**A-B**), or a Western-type cholesterol-rich diet (**C-D**). The SAF84 antibody was used to detect PrP^{Sc} proteins and the Vectastain ABC-AmP Kit (Vector laboratories, USA) to reveal antibody binding.
