# Supplementary material for: Plasma cholesterol level determines in vivo prion propagation
Source: J Lipid Res. 2017 Aug 1;58(10):1950–61. doi: 10.1194/jlr.M073718 (PMC5625119; doi:10.1194/jlr.M073718)
Supplement: Supplemental Data [file 10.1194_M073718_jlr.M073718-2.pdf]

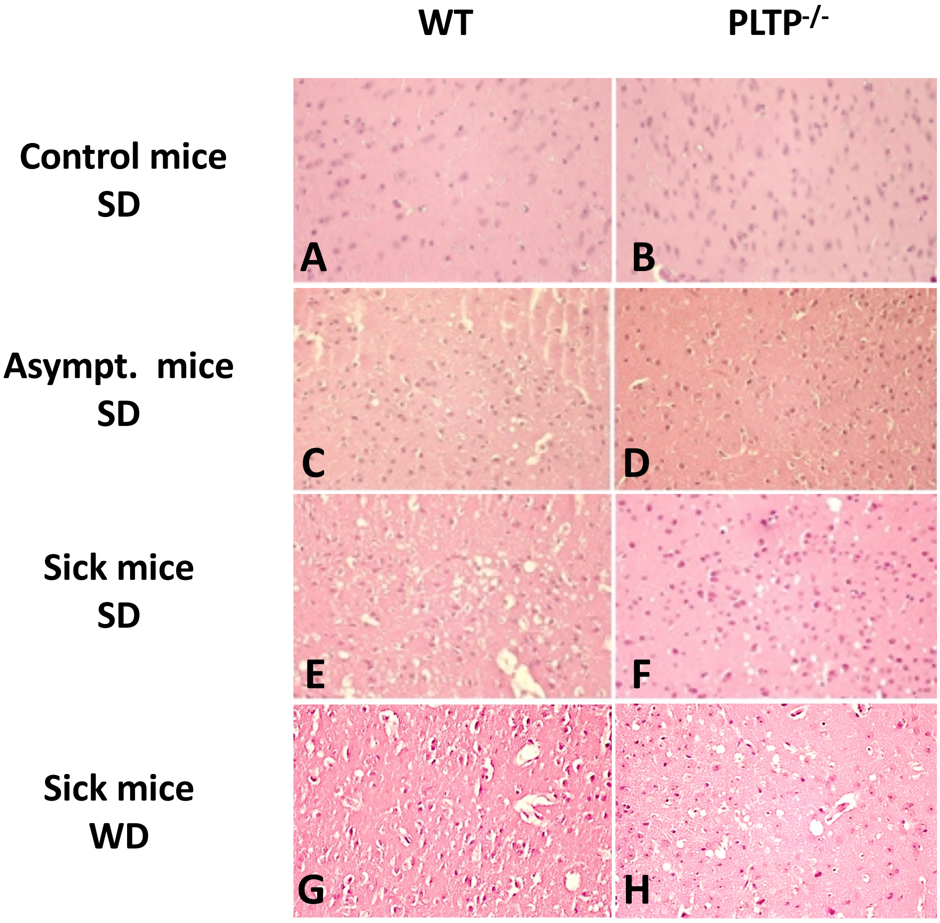

Supplemental Figure S2. Analysis of spongiosis in brain tissue sections.
(**A-B**) Histological analyses of thalamus (Th) sections from control WT and PLTP$^{-/-}$ mice not inoculated with prions. (**C-H**) Thalamus tissue sections from WT and PLTP$^{-/-}$ mice inoculated with 22L prion strain and either sacrificed while they were asymptomatic (196 d.p.i) (C-D), or at the terminal stage of the disease (E-H), and fed either a standard chow diet (SD) (E-F), or a Western-type cholesterol-rich diet (WD) (G-H). Tissue sections were stained with hematoxylin and eosin (H&E) to confirm the prion disease as indicated by the presence of vacuoles.Tissue labeling was performed on several animals (2-3 per group, except asymptomatic mice) and the selected images are representative of the staining observed in each group.
